# Personal Exposure Assessment of Respirable Particulate Matter Among University Students Across Microenvironments During the Winter Season Using Portable Monitoring Devices

**DOI:** 10.3390/toxics13070571

**Published:** 2025-07-07

**Authors:** Muhammad Jahanzaib, Sana Iqbal, Sehrish Shoukat, Duckshin Park

**Affiliations:** 1 Korea Railroad Research Institute (KRRI), Uiwang-si 16105, Republic of Korea; jahanzaib@ust.ac.kr (M.J.); sehrish@krri.re.kr (S.S.); 2 Transportation and Environment Research Centre, Korea University of Science and Technology, Daejeon 34113, Republic of Korea; 3 Institute of Environmental Science and Engineering (IESE), School of Civil and Environmental Engineering (SCEE), National University of Sciences and Technology (NUST), H-12, Islamabad 44000, Pakistan

**Keywords:** air pollution, respirable particulate matter, particle number count, microenvironment, real-time monitoring

## Abstract

Respirable particulate matter (RPM) is a major indoor environment concern posing direct health risks. Localized data on RPM exposure remains scarce across different microenvironments in occupational and educational settings. Students in educational settings are increasingly vulnerable to RPM, specifically in the winter season when more activities are carried out indoors and meteorological conditions elevate the PM levels. This study was conducted to assess the personal exposure of university students to RPM within their frequently visited microenvironments (MEs). Forty volunteers were selected, and their exposure to RPM was measured by specifically monitoring their particle mass count (PMC) and particle number count (PNC) in commonly identified MEs. Calibrated air pumps with nylon cyclones and a Dylos DC 1100 Pro were used for this purpose. We found that the mean RPM concentration for personal exposure was 251 µg/m^3^, significantly exceeding the prescribed National Environmental Quality Standards (NEQS) limit of 35 µg/m^3^. We also observed a significant correlation between the PNC and PMC in the microenvironments. The assessment of personal exposure to RMP in this study highlights the urgent need for mitigation strategies in educational settings to reduce the personal exposure of students to RMP to reduce their health-related risks.

## 1. Introduction

Rapid industrialization, growth, and urban advancements have come with the cost of compromised air quality. Particulate matter (PM), considered a primary air pollutant, remains suspended in the air [1] and poses a significant concern to human health due to its deep penetration ability into the respiratory system, affecting short-term and long-term well-being [2,3]. Epidemiological studies have linked severe health outcomes to PM exposure, including morbidity and mortality [4,5]. According to the World Health Organization, 4.2 million global deaths in 2021 were related to PM exposure [6] (WHO, 2021). The fractional size of PM is the major factor that determines its health impact. Respirable particulate matter (RPM), having a particle size < 4 µm in aerodynamic diameter, is hazardous due to its ability to infiltrate into the lungs [7]. Personal exposure to PM is defined as the measurement of a fraction of PM near the breathing zone [8]. It varies with an individual’s activity pattern and the specific microenvironments they occupy, such as indoor and outdoor settings [9]. The study of [10] on university students’ exposure to PM_2.5_ determined that students spend ~40% of their time in classrooms, ~50% in dormitories, ~5% in commuting, and ~5% in cafeterias. This personal exposure is highly influenced by multiple factors, such as indoor contributors, including cooking and smoking, while outdoor contributors include traffic and industrial emissions. This highlights the need for detailed assessments of personal exposure in microenvironments to understand their PM exposure risk effectively and devise strategies and devices to mitigate this effect [11].

Microenvironments are localized areas where individuals conduct their daily activities, such as homes, classrooms, workplaces, and dormitories, and are exposed to air pollutants, including RPM [11]. Microenvironments are critical to study because of their direct influence on human health, leading to severe respiratory and cardiovascular diseases [12]. The time spent in a specific microenvironment shapes one’s personal exposure to RPM as the pollutant varies in each setting [13]. Different studies show that individuals spend 70–80% of their time indoors [14,15], where RPM levels can significantly differ due to different sources, occupancy, and ventilation than in an outdoor setting [9]. A recent study [16] shows 2–3 times higher PM_2.5_ concentrations in indoor classrooms with poor ventilation than outdoor levels, showing that students were exposed to such high concentrations for 5–6 h in such settings. Conversely, routes and places near heavy traffic can spike for shorter periods during rush hours, with PM_4_ concentration reaching 300 µg/m^3^, as noted by [17]. In the study of [8], different workplaces were assessed to estimate RPM exposure, specifically focusing on personal exposure to PM_4_, finding a maximum concentration of 286 µg/m^3^ for a hairdresser exposed to aerosols and hair sprays, followed by an optics shop employee having exposure to 31 µg/m^3^ of RPM. A housewife experienced the lowest exposure, with an RPM concentration of 27 µg/m^3^. These variations show a necessity to study the time spent in microenvironments to understand the exposure patterns for targeted interventions to reduce RPM’s health risks, particularly in high-exposure settings such as educational campuses and workplaces [1]. Moreover, multiple factors such as the age, gender, lifestyle, working conditions, and socioeconomic status of an individual modulate personal exposure in the same microenvironment [18], emphasizing the need for personalized exposure assessments to protect the vulnerable population.

Personal exposure to PM concentrations is greatly influenced by seasonal variations [19], with winters exhibiting higher concentrations of PM due to temperature inversions and elevated emissions from heating sources [20]. A study by [21] used trained technicians who followed scripted time–activity patterns during the summer and winter seasons, involving 2358 inhabitants of Seoul (South Korea), and reported elevated concentrations of PM in winter as compared to summer. They found that the mean daily PM_10_ concentration of 37.8 ± 25.6 μg/m^3^ in summer reached 48.5 ± 32.9 μg/m^3^ in winter. Similarly, the average daily exposure to PM_2.5_ was 27.8 ± 21.4 μg/m^3^ and 36.9 ± 28.7 μg/m^3^ in the summer and winter seasons, respectively, whereas the highest value of PM_2.5_ exposure went up to 59.8 ± 52.3 μg/m^3^. Also, personal exposure was non-significantly higher in winter (*p* = 0.108) as compared to summer. The study in [18] investigated the seasonal variations in fine particulate matter in different households’ indoor microenvironments in Lahore, finding the highest mean level of fine PM in winter, followed by the monsoon and summer seasons, respectively. The measurement of PM in air is commonly carried out via filter-based sampling, which collects particles on filters for gravimetric analysis, and real-time PM monitors are also used, which provide instantaneous data [22,23]. Most of the sensors work upon the light scattering principle, a method widely adopted for its efficiency and sensitivity [24]. However, in recent studies, scientists have validated the use of low-cost sensors, a cost-effective alternative to traditional methods for monitoring personal exposure to PM [19]. Multiple studies have used low-cost sensors to quantify PNC for personal exposure and to quantify airborne RPM in different microenvironments [25]. In the study by [4], a user-friendly particle counter (Dylos DC 1700) was used for short-term personal exposure assessment in different microenvironments. The authors measured the PNC in homes (186,444 ± 415,535 ft^−3^), private residential building (228,898 ± 366,312 ft^−3^), public buildings (135,632 ± 202,852 ft^−3^), workplaces (55,687 ± 52,194 ft^−3^), outdoors (147,444 ± 201,028 ft^−3^), and transport (151,428 ± 202,423 ft^−3^) [4]. This approach aligns with the methodology of the current study to measure the PNC and PMC in university microenvironments, revealing a correlation between these metrics and underscoring the utility of low-cost sensors in studies related to PM exposure.

The necessity of this study stems from the urgent need to understand microenvironmental exposure among university students who spend significant time in indoor settings like classrooms, where the PM level is higher than in outdoor settings due to poor ventilation. Moreover, the winter season exacerbates exposure risks, posing serious concerns to human health. Given the significant health risks imposed by RPM exposure across microenvironments, the present study assesses the personal exposure to respirable particulate matter of students on a university campus across different microenvironments during the winter season. The primary objectives of this study were to (i) comprehensively assess the personal exposure to RPM by quantifying concentration through simultaneous measurement of PMC (particle mass count) and time-resolved PNC (particle number count) near breathing zones during the winter season, and (ii) evaluate the contribution of specific microenvironments—such as classrooms, dormitories, cafeterias, and commuting routes—to overall RPM exposure, addressing a gap in previously reported studies, which often overlooked the role of individual microenvironments in cumulative exposure.

## 2. Materials and Methods

### 2.1. Study Area and Subjects

This study was conducted at a public sector university. The university campus spans an area of 707 acres and is home to approximately 3000 students.

The campus is surrounded by significant sources of air pollution, including major roads and ongoing construction activities. To the northeast of the campus lies a highway, a 10-lane road with heavy traffic flow, serving as a major source of vehicular emissions. Construction activities related to the metro bus extension project along the highway added to the pollution levels during the sampling period, which lasted from November to February.

To the southwest of the campus is Inter Junction Principal Road (I.J.P.), which connects two cities. This road experiences heavy traffic, including various types of vehicles such as cabs without catalytic converters, heavy container trucks, and rickshaws. Additionally, a residential sector situated nearby was undergoing development, with the construction of high-rise apartments, contributing to PM pollution.

Furthermore, an industrial hub located a few kilometers away from the university hosts several industries, including marble, steel, aluminum, and pharmaceutical production. Emissions from these industries impact the ambient air quality of the city [14]. Further, Figure S1 in the Supplementary Information provides information regarding the study site, and Figure S2 in the Supplementary Information shows the methodological layout of this study.

### 2.2. Personal Exposure Monitoring

Personal exposure monitoring of RPM was conducted to assess the exposure level of university students in various microenvironments during the winter season, a period in which the PM concentration is high due to meteorological conditions.

#### 2.2.1. RPM Mass Concentration (RPMMC) Monitoring

**Figure 1:**
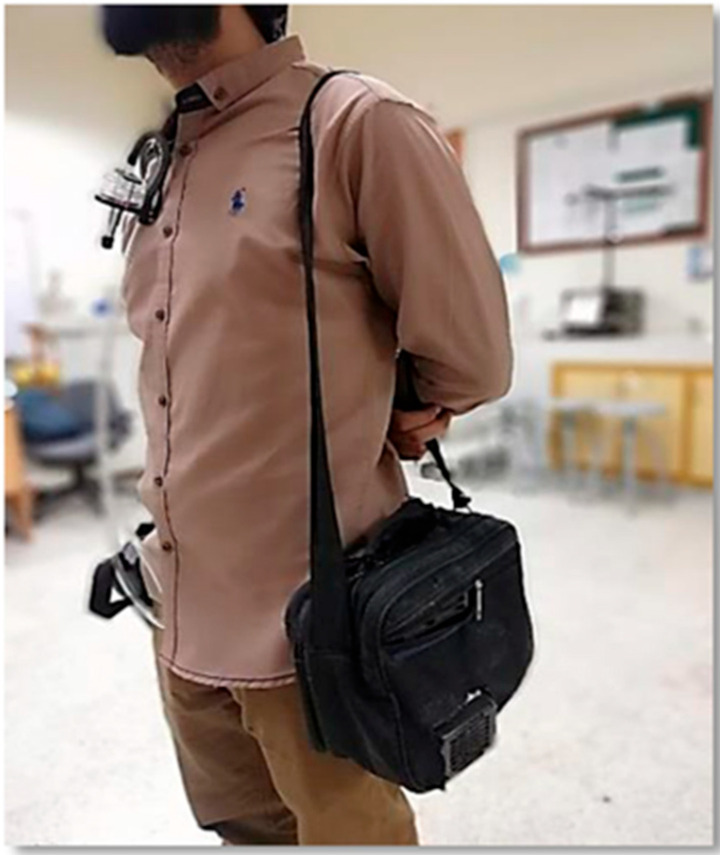
Cyclone in the breathing zone of a volunteer with a Gillian 5000 suction pump.

Sampling took place over three months, from November to January, during regular working hours, to assess the RPMMC among university students. A total of 40 volunteer students were randomly selected for this study. RPMMC samples were collected from the breathing zone of each volunteer (See Figure 1), with one sample taken from each individual to ensure consistency in exposure assessment. Each participant was monitored individually on separate days to capture their representative exposure under typical daily conditions. Mixed cellulose ester filter paper (37 mm diameter, Zefone MCE) was utilized for RPM mass sampling. A sampling cassette for the filter paper (37 mm) was attached to a conductive nylon cyclone (Zefone). A personal air sampler (Gillian 5000 Pump-Sensidyne, FL, USA) was employed to draw air into the sampling cassette at a calibrated flow rate of 1.7 L/min [26,27], following protocols for respirable dust sampling [28] (NIOSH, 2016). Flow rate calibration was conducted before and after the collection of each sample to ensure stability and accuracy throughout the collection process. Similarly, the personal air sampler was calibrated before and after the collection of every single sample. To mitigate the influence of environmental factors, the MCE filter paper was stored in a clean, air-tight container both before and after sampling and maintained in a controlled environment with a controlled humidity and temperature to prevent moisture- and temperature-related biases in the PM measurements. Following the Environmental Protection Agency (EPA) recommendation for quality assurance (1999), a blank filter paper was included with every batch of 20 filter papers to account for potential contamination and ensure data integrity.

##### Gravimetric Analysis

A gravimetric analysis of the RPM was performed to determine the mass concentration of the collected samples following the standardized protocols [29] (EPA 1999). The pre- and post-weights of MCE filters having a diameter of 37 mm were measured using a high-precision electronic microbalance (X.A.6.4YM, Radwag, FL, USA), with an accuracy limit of 1 µg, ensuring the accurate quantification of the particulate mass. Each cellulose filter paper was weighed three times to obtain the average value before and after each sample collection. The filter papers were conditioned in a controlled environment to eliminate moisture-related biases during the measurements. The gravimetric analysis for RPM μg/m^3^ (following EPA guidelines (1999)) was calculated using the following formula:

RPM = (W_f_ − W_i_) × 10^6^/V (1)

where

W_i_ is the initial weight;

W_f_ is the final weight of the filter paper;

10^6^ is the conversion of a gram to a microgram; and

V is the total volume of the air (m^3^) pulled through the cyclone.

#### 2.2.2. RPM Number Count (RPMNC) Monitoring

The RPMNC, also known as PNC in this study, was monitored to complement the mass-based measurements of RPM and provide a comprehensive insight into the personal exposure in different microenvironments among university students. A Portable Dylos DC 1100 Pro (Riverside, CA, USA) was utilized in this study, which is a low-cost particle counter weighing 1.2lb and equipped with an on-screen digital display. It measures particle count in two sizes of bins (>0.5 and >2.5) per minute interval using light-scattering technology [30]. Although originally designed for indoor air quality monitoring, the Dylos has been successfully validated and employed in both indoor and outdoor applications (e.g., [21,31]). In our research to capture the minute-by-minute PNC in the breathing zone of the participants, the Dylos DC 1100 Pro was integrated with a personal air sampler, necessitating the development of a customized portable setup (Figure 1). Due to the absence of a built-in battery in the Dylos, continuous power was supplied by a 12 V lead–acid battery, which was replaced every 12 h to ensure uninterrupted operation during the sampling periods. Data logging was facilitated by software provided by the Dylos Corporation (Riverside, CA, USA), with portable logging achieved using a Raspberry Pi 3, enabling real-time data collection and storage.

The instrumental setup was housed in a specifically designed bag, ensuring the direct exposure of the Dylos inlet and outlet to ambient air while securely enclosing the battery and Raspberry Pi 3 inside the bag (Figure 1) [4,32]. This setup configuration enabled the simultaneous collection of RPMNC data alongside the RPMMC data from volunteers, providing a comprehensive exposure profile. Due to limited instrument availability and battery constraints with the suction pump (Gillian 5000 pump Sensidyne, FL, USA), RPMMC monitoring was limited to 8 h. In contrast, the battery limitation for the Dylos was resolved by using rechargeable batteries, extending RPMNC monitoring to 20 h per session, thus capturing a more complete diurnal exposure pattern for the participants.

RPMMC and RPMNC were reported separately. The RPMMC data were reported according to 8 h. But for correlation, the RPMMC and RPMNC data were taken for 8 h for precise analysis. The personal air sampler (Gillian 5000 Pump) had a built-in battery that only worked for 8 h, and the recharging time was about 4 h. So, it was not feasible to perform gravimetric sampling for 22 h. Due to this limitation, sampling was conducted during the university’s working hours. For more precision, a microenvironment gravimetric analysis was conducted.

#### 2.2.3. Temporal Activity Distribution Analysis

To characterize the time–activity patterns of the volunteers across each microenvironment, each individual was provided with a detailed time–activity diary. Volunteers were instructed to record their entry and exit times for each microenvironment along with detailed notes on their activities every 15 min. This approach, widely used in exposure studies, enabled the precise allocation of time spent in different settings, which is an important factor in understanding microenvironmental contributions to overall exposure [9]. Subsequent post-sampling interviews were conducted to clarify any ambiguities and validate the recorded data for each sample, following the best practices for data quality assurances [33]. Time spent within a microenvironment (ME) was classified as indoor, while the remaining time was categorized as non-indoor, which included both in-vehicle and various outdoor MEs. It is important to note that in-vehicle environments, such as inside cars, buses, or trains, differ substantially from general outdoor settings due to their confined space, distinct ventilation characteristics, and proximity to direct emission sources. Moreover, outdoor MEs themselves are heterogeneous, encompassing diverse settings such as markets, parks, roadside areas, and open urban spaces. To validate the accuracy of the location data and distinguish between indoor and outdoor microenvironments, a GPS receiver (Globalsat DG-200- Global Sat WorldCom Group, Taipei, Taiwan) was affixed to the instrument enclosure carried by each participant. The GPS data logged continuously during sampling were cross-referenced with the time–activity diary entries to confirm the spatial accuracy of the reported locations, enhancing the reliability of the microenvironment exposure assessment. This dual validation method ensured the precise mapping of time–activity patterns, facilitating an accurate evaluation of RPM exposure in the breathing zone across diverse microenvironments.

### 2.3. Microenvironment Identification and Monitoring

The time–activity diaries completed by every participant were thoroughly reviewed and analyzed to identify the most frequented microenvironments (MEs) during the winter season. This systematic review enabled the identification of key MEs influencing personal exposure to RPM, which is a critical step in understanding the exposure variability across different settings. Among the various MEs documented, the most prevalent MEs included hostel rooms, hostel messes, open cafés, indoor cafés, laboratories, and classrooms, later categorized as with or without Heating, Ventilation, and Air Conditioning (HVAC) systems, showing diverse indoor air quality conditions. These MEs were selected based on their high occupancy rates and activity patterns, which significantly affect RPM levels. Subsequently, RPMMC and RPMNC monitoring was conducted across all eight MEs for a continuous 24 h period per microenvironment.

Buildings equipped with HVAC systems demonstrate controlled indoor environmental conditions, maintaining air changes per hour (ACH) between 6 and 12, ventilation rates of 10–15 L/s per person, and carbon dioxide (CO_2_) concentrations consistently below 1000 ppm. These systems also regulate temperature within 20–24 °C and relative humidity between 30 and 60%, while significantly reducing both RPMMC and RPMNC through high-efficiency filtration (e.g., MERV 13 or higher). In contrast, non-HVAC (naturally ventilated) buildings exhibit a variable ACH ranging from 0.5 to 3, inconsistent ventilation rates, and elevated CO_2_ levels often exceeding 1200 ppm, with higher and uncontrolled RPMMC and RPMNC levels due to the absence of mechanical filtration and reliance on external environmental conditions for air exchange.

#### 2.3.1. RPM Mass Concentration (RPMMC) Monitoring

Microenvironment monitoring was conducted from November to January to assess the RPM levels across various settings frequented by university students, with each sample collected over 24 h to capture diurnal variations in exposure. Air samplers were strategically positioned at the average breathing zone height within each ME to ensure a representative measurement of inhalable RPM. For RPM mass collection, ester cellulose filter paper (37 mm diameter, Zefone MCA) was combined with a Nylon cyclone (Zefone) to selectively capture respirable particles. A portable air suction pump was employed to draw in air at a flow rate of 2.5 L/min, adhering to the standardized protocols for respirable dust sampling [28] (NIOSH, 0600). In total, 18 samples were collected from various identified microenvironments, with 5 samples from hostel rooms (including cubicle or single-seater, bi-seater, and tri-seater configurations) and 2 samples from each of the other microenvironments (open café, indoor café, laboratory, and classrooms with and without HVAC systems). The limited number of sample constraints was due to equipment availability. Additionally, a higher sample count would have extended the sampling duration beyond the sampling period, potentially introducing seasonal variability as a confounding factor. Therefore, to mitigate this, efforts were made to prioritize both personal exposure and ME monitoring within the winter season, ensuring consistency in meteorological conditions.

#### 2.3.2. RPM Number Count (RPMNC) Monitoring

RPMNC monitoring across all identified microenvironments was conducted using the same instrumental setup developed for personal exposure assessment, which ensured the meteorological conditions and enabled us to compare data consistently. The setup previously mentioned for personal monitoring utilized the Dylos DC 1100 Pro to measure PNC in two sizes (>0.5 µm and >2.5 µm) at 1 min intervals alongside a portable data logging system with a Raspberry Pi 3. Both the RPMNC and RPMMC setups were installed adjacent to each other to ensure consistency. The duration of RPMNC monitoring was 24 h in all eight microenvironments.

### 2.4. Statistical Analysis

To evaluate the relationship between the real-time particle number count and gravimetric mass concentration of both the personal exposure and microenvironment samples, correlation coefficient analyses were performed, which are widely used to observe linear relationships in air quality studies. Basic statistical analyses and correlation tests were carried out using MS Office Excel 365 and Minitab 16. Statistical analyses were performed with a 95% confidence interval and a significance threshold level of α = 0.05, following standard practices. Additionally, to assess the difference in RPMMC exposure between microenvironments equipped with HVAC and those without HVAC, a *t*-test was employed, aiming to identify the impact of ventilation on exposures across various settings.

### 2.5. Precipitation Data

Data for precipitation was collected for the sampling duration, i.e., from November to January, and was obtained from the historical average weather data provided by worldweatheronline.com (https://www.worldweatheronline.com). Rainfall data is necessary to identify the effect of rainfall on the PM concentration in the air, as it critically influences particle dynamics via wet deposition. Low rainfall in winters can exacerbate PM accumulation, leading to higher RPM levels. The incorporation of precipitation data in this study ensured meteorological variability, enabling the accurate study and interpretation of RPM exposure patterns posing health implications across various microenvironments in educational settings [34]. Weather variability significantly affected the measurements; during rainy days, the RPMMC and RPMNC values were recorded as low, and during windy and stormy days, the recorded measurements were high. The figure for the mean monthly precipitation data during the months of this study is provided in the Supplementary Materials (Figure S3).

## 3. Results and Discussion

### 3.1. Personal Exposure Measurements

The personal exposure to RPM among 40 volunteer university students was assessed during the winter season (November to January), with the statistical results summarized in Table 1.

#### 3.1.1. RPMNC and RPMMC Measurements

The mean RPMMC, measured using gravimetric analysis, was 251 µg/m^3^ (±91 µg/m^3^), and the mean RPMNC was 175 m^−3^ (±84 m^−3^). These results reflect the substantial variability in particle exposure across the students and days. December recorded the highest mean values for both RPMNC and RPMMC, while January had the lowest, suggesting a strong influence of meteorological conditions on PM exposure.

##### Temporal and Meteorological Influences on RMP Levels

**Figure 2:**
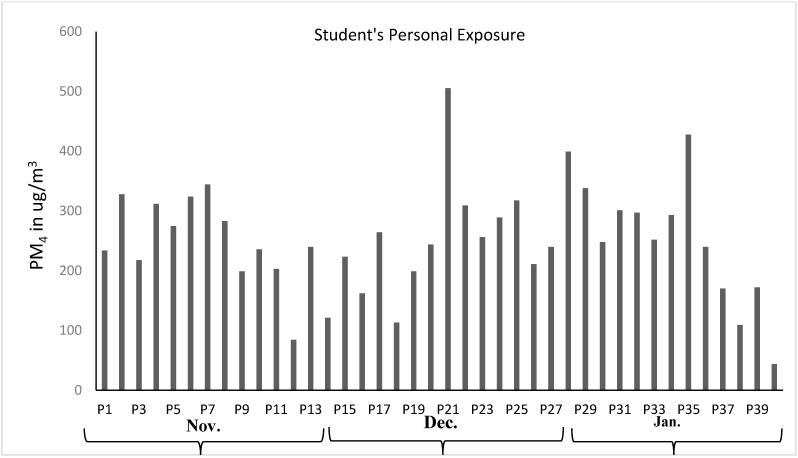
Respirable particulate matter mass concentrations observed from personal exposure of students (µg/m^3^) and comparison between concentration levels of RPM in November, December, and January.

**Figure 3:**
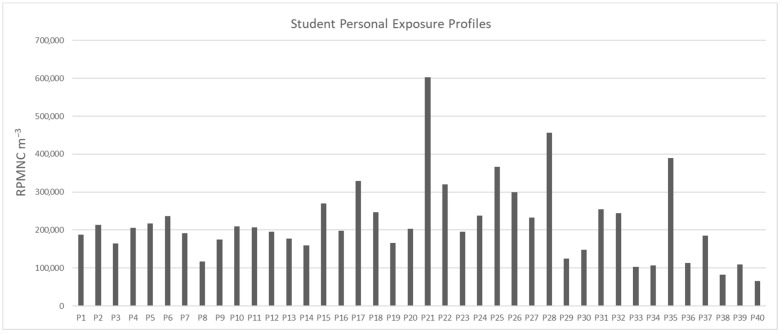
Respirable particulate matter number count from personal exposure of students (m^−3^) and comparison between concentration levels of RPMNC in November, December, and January.

The lowest recorded RPMNC and RPMMC were 52 m^−3^ and RPMMC 43 µg/m^3^, respectively, observed on a full rainy day at the end of January, corresponding to Profile 40 (Figure 2) and Figure 3, reflecting a significantly reduced PM level compared to this study’s mean values of 251 µg/m^3^ and 175 m^−3^ for RPMNC and RPMMC, respectively. This decrease is attributed to the washout effect of precipitation, a well-documented phenomenon for the removal of PM from ambient air through wet deposition, which lowers the atmospheric PM concentration [35].

Precipitation data from the meteorological department indicated higher rainfall in January as compared to November and December, with the majority of rainfall events concentrated at the end of January. Consequently, the profiles measured during this period exhibited notably lower RPM levels, aligning with monthly trends showing the lowest mean RPMNC and RPMMC in January and the highest in December. These findings are consistent with prior studies in South Asia, where reduced PM levels were reported during wet periods due to enhanced particle scavenging [34]. The observed influence of precipitation underscores its role as a critical meteorological factor in modulating PM exposure for university students, particularly in microenvironments where ambient PM levels directly impact personal exposure.

In Islamabad, Pakistan, December and January are winter months, characterized by the lowest annual temperatures, a climatic pattern that significantly influenced air quality during the sampling periods. This study observed the highest monthly mean RPMNC and RPMMC in December, with values substantially elevated as compared to January, reflecting the influence of winter meteorological conditions on PM levels. The increased PM levels in the South Asia region align with many prior studies that highlight winter conditions, including temperature inversions and reduced mixing height, trapping PM in ambient and indoor microenvironments [36].

The weather pattern in December and January typically brings the lowest temperatures to Islamabad due to the winter season. Numerous studies have reported higher concentrations of ambient and indoor PM levels during winter compared to the summer months. This phenomenon is attributed to the reduced dispersion of PM and increased suspension due to lower temperatures, ultimately leading to poor air quality. Consistently, the highest RPMMC and RPMNC (monthly mean) were measured in December, reflecting the impact of winter weather conditions on PM levels.

##### Comparison with Air Quality Standards

The mean RPMMC of 251 µg/m^3^ exceeded the National Environmental Quality Standards (NEQS) of Pakistan’s Environmental Protection Agency (EPA) for ambient PM_2.5_ (35 µg/m^3^) and PM_10_ (150 µg/m^3^) over 24 h [37]. Even the minimum RPMMC of 44 µg/m^3^, recorded over an 8 h sampling period, surpassed the NEQS PM_2.5_, indicating high exposure levels for students, even on days with precipitation-induced reductions.

For RPM (PM_4_), no specific limit has been specified by NEQS, EPA Pakistan. However, the American Council of Governmental Industrial Hygienists (ACGIH) and the U.S. Occupational Health and Safety Administration (OSHA) specify the permissible exposure limits of 3 mg m^−3^ and 5 mg m^−3^, respectively, for respirable dust over 8 h [19,24].

All calculated values of RPMMC in this study were lower than the permissible limit of ACGIH and OSHA; however, these occupational standards are designed for a workplace setting and may not be adequate for educational microenvironments for vulnerable populations like students, who are susceptible to high PM levels in universities. The exceedance of the NEQS ambient standards, coupled with the mean RPMMC far above the WHO guidelines of 25 µg/m^3^, shows the dire need for air quality interventions in a university setting to protect students’ health and mitigate personal exposure to RPM. Table 2 shows the monthly mean value of RPMMC in µg/m^3^ and RPMNC in m^−3^ of personal exposure of students in an educational setting.

##### Temporal Profile Distribution and Analysis

**Figure 4:**
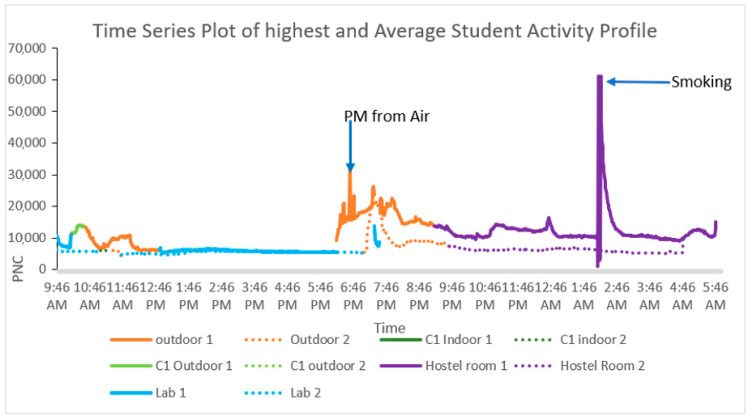
Time–activity plot of the two students exposed to the highest RPMNC (continuous line) and an average RPMNC (dotted line). Different MEs are shown with different colors.

The time–activity profiles of two different students, selected based on their RPMNC exposure levels, are illustrated in Figure 4, highlighting the influence of MEs and the activities carried out. These profiles were selected based on the highest PNC (483 m^−3^) and the average PNC (174 m^−3^) recorded. Student 1 (profile 21, Figure 3), represented by a solid line, exhibited the highest RPMNC of 483 m^−3^ over a 20 h monitoring period, while student 2 (profile P5, Figure 3), depicted by a dotted line, showed a near-average RPMNC of 174 m^−3^, closely aligning with this study’s mean value of 175 m^−3^ (Table 1). Monitoring commenced at 09:46 a.m., with the instrumental setup (Dylos DC 1100 Pro) provided to both students, who then proceeded to their respective laboratories for research work. In the time spent in the laboratory, both students recorded similar average RPMNC levels of 171 m^−3^ (Figure 4), mainly due to their comparable activities under identical ventilation conditions. Good ventilation can likely minimize the particle resuspension, hence less personal exposure to RPM [38]. Student 1 subsequently visited an open-air café at 10:05 am, spending 19 min there, during which the average RPMNC value reached 372 m^−3^, reflecting elevated outdoor PM exposure, likely due to dust and emissions present in ambient outdoor air. In contrast, student 2 visited an indoor café at 11:06 am, experiencing a lower average RPMNC of 171 m^−3^, indicating better air quality due to being indoors and reduced external PM infiltration.

Both students exhibited notable fluctuations in RPMNC during their outdoor activities, particularly during walking, as shown in Figure 4, indicating varying levels of PM exposure in ambient conditions. In outdoor conditions, the primary source of PM exposure was the ambient air. As both volunteers were constantly moving, their exposure levels to PM varied accordingly. Student 1’s profile recorded the highest RPMNC peak during outdoor walking conditions. Also, student 1 experienced the second-highest peak, observed at 02:12 am, attributed to second-hand smoke from a cigarette during a group study session in a poorly ventilated indoor setting. On the sampling day, closed windows and low ventilation, which are common in winter to retain heat, led to exacerbated indoor smoke accumulation, increasing the RPMNC.

In contrast, on student 2’s sampling day, there were no group gatherings, and they were a non-smoker, resulting in a different exposure profile with lower indoor peaks. These findings show the significant impact of activity patterns and MEs on PM exposure, emphasizing the need for improved ventilation in indoor education microenvironments.

### 3.2. RPMMC and RPMNC Measurements Across Microenvironments (MEs)

#### 3.2.1. RPMMC Levels in Indoor and Outdoor Café MEs

A descriptive statistical analysis of the MEs monitored revealed distinct patterns in the RPMNC and RPMMC across the various settings frequently visited by university students during the winter season. The overall mean RPMNC across all MEs was 87 m^−3^ (n = 18), with a maximum of 523 m^−3^ observed in a classroom without an HVAC system, mainly due to its high occupancy and poor ventilation rate, whereas the minimum RPMNC of 31 m^−3^ was observed in a lab without HVAC, possibly reflecting its lower occupancy and fewer particle generation activities. The RPMMC results show a mean concentration value of 69 ± 51 µg/m^3^. In this case, the maximum and minimum concentrations of 186 µg/m^3^ and ~10 µg/m^3^ were obtained in an indoor café and the hostel cubicle room, respectively (Table 3). According to the average time–activity patterns of the volunteers in this study, most of their time was spent in school MEs rather than hostel rooms.

**Figure 5:**
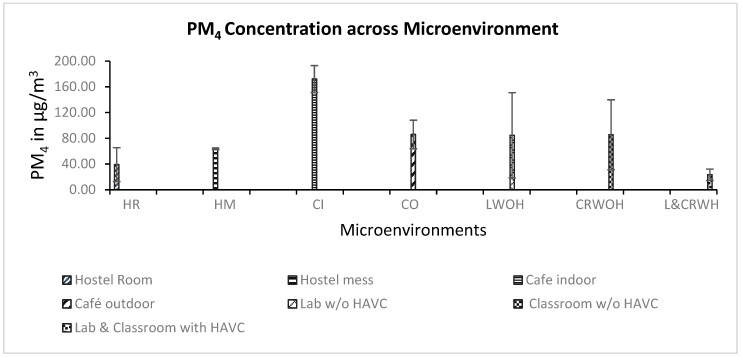
Average RPMC of all MEs with their standard deviation.

The average PMC values across all microenvironments are shown in Figure 5, with the highest PMC value measured from the indoor café, 172 ± 2 µg/m^3^, exhibiting notably high concentrations. The high PM concentration observed in the indoor café microenvironment has several reasons, primarily, being one of the main cafés on the university campus, attracting a large crowd, particularly during break times when occupancy and food-related emissions are high because the café’s kitchen is connected to the sitting area, contributing to PM emissions. Moreover, in addition to the café’s adjacent kitchen, the presence of a live BBQ station near the entrance adds to its PM sources, as fumes and smoke from the BBQ elevate the PM concentration in the indoor air in this specific microenvironment. Furthermore, eating and drinking are the primary activities conducted in this microenvironment, which can further elevate indoor PM concentrations through the dispersion of food-related particles and increased occupant movement. It is worth noting that the indoor PM concentration in any microenvironment is directly influenced by the type of activities conducted within it. Cooking and the presence of persons at a specific ME lead to an increase in RPM level [39]. The second highest concentration, with an average RPMMC of 86 ± 16 µg/m^3^, was measured in the outdoor café, which features an open-sided design with a covered roof, facilitating higher ventilation rates as compared to the indoor café. This enhanced ventilation likely reduced PM accumulation, significantly resulting in a lower RPMMC than in the indoor settings, consistent with the findings of [40], which states that the indoor air quality is influenced by the ventilation in semi-open MEs.

#### 3.2.2. Impact of HVAC Systems on RPMMC in Laboratories and Classrooms

The monitoring of RPMMC in laboratories and classrooms equipped with HVAC systems in their MEs showed different results as compared to labs and classrooms without HVAC systems during the winter season. The lower RPMMC was measured in the laboratories and classrooms that had an HVAC system as compared to non-HVAC. In terms of statistical measurement, a *t*-test was performed to analyze the variance between the two data sets. The *p*-value (*t*-test) was >0.05; therefore, the difference in the values of the RPMC in the HVAC and non-HVAC systems varied non-significantly. The average RPMMC in the labs and classrooms without HVAC was (85 ± 47 µg/m^3^) and (86 ± 38 µg/m^3^), respectively, and with HVAC RPMMC was (23 ± 4 µg/m^3^) (Figure 5), indicating the improved air exchange in HVAC systems, reducing indoor PM concentration. The substantially low PM levels in HVAC-equipped MEs show the practical importance of ventilation systems in mitigating PM exposure in educational settings for university students who spend most of their daily time in such environments [41].

The hostel room (HR) and hostel mess (HM) represent other critical MEs for students, as they are where they spend most of their time after studies. The average RPMMC measured in the HR was 39 ± 12 µg/m^3^, notably lower than the 64 ± 1 µg/m^3^ recorded in the HM, reflecting the distinct PM sources and activity patterns in these settings. The higher RPMMC level in the HM as compared to the HR could be due to increased occupancy and activities such as eating and drinking, which affect PM concentration; however, in the HR, the RPMMC source is ambient air, and also comes from personal hygiene products (cosmetics) and smoking.

### 3.3. Correlation Between RPMMC and RPMNC

The relationship between RPMMC and RPMNC was evaluated for both personal exposures and MEs using Pearson correlation analysis, with the strength of the linear relationship indicated by the coefficient of determination (R^2^). RPMMC was determined by gravimetric analysis, while RPMNC was measured with a Dylos DC 1100 Pro. A high R2 value (closer to 1) indicates a strong linear relationship, showing that sources contributing to the particle mass also contribute to particle counts. In this study, the correlation analysis revealed a notable positive correlation (*p* < 0.05) between the RPMMC and RPMNC across personal exposures and MEs.

A strong positive correlation, with R approaching 0.75, was observed between the microenvironment data of the RPMMC and RPMNC, indicating that ~56% of the variability in the RPMMC could be explained by the RPMNC (R^2^ = 0.56), with *p* = 0.0006. This linear regression model yielded the following relationship (Equation (2)) with 95% confidence. This indicates that ~56% of the variability in particle mass can be explained by the particle number in microenvironmental settings such as classrooms or kitchens.

(2) y=21.205x+1447.6

This relationship shows that PM sources in MEs, such as cooking in an HM or occupancy in classrooms, contribute to both the mass and number count of RPM. These correlation results are found to be aligned with the findings of [42].

However, the correlation between RPMMC and RPMNC in personal exposure was found to be weaker, with an R-value of 0.41, R^2^ = 0.17, and *p* = 0.089, implying greater variability in exposure dynamics as students traveled between MEs, like commuting routes and indoor settings. The corresponding regression equation is Equation (3), with a 95% confidence interval. This weaker association likely reflects the greater variability encountered during daily activities as participants moved across diverse microenvironments.

(3) y=13.808x+3148.8

The study by [24] also shows a similar disparity due to the influence of diverse and transient sources and activity patterns, which ultimately affect personal exposure. This shows that outdoor setting conditions are less consistent than controlled ME conditions.

The Dylos instrument, originally designed for indoor particulate monitoring, was adapted for portable use in this study to measure the RPMNC in MEs and for personal exposure monitoring during the winter season. While the instrument maintained its stability during ME monitoring, yielding a strong correlation between RPMMC and RPMNC, its performance was compromised during personal exposure monitoring due to the dynamic movement of the volunteers between different microenvironments as part of their daily routines. This instability due to motion and varying environmental conditions might have caused a weaker correlation observed in the RPMMC and RPMNC in personal exposure compared to the microenvironments.

To further validate the relationship between RPMMC and RPMNC, a linear regression model was applied, revealing significant variations between the two metrics in both MEs and personal exposure, where *p* values were <0.05, showing that factors beyond the correlation line influenced the observed difference.

## 4. Conclusions and Recommendations

This study meticulously assessed the personal exposure of university students to respirable particulate matter mass concentration (RPMMC) and respirable particulate matter number concentration (RPMNC) within their campus environment, complemented by a detailed evaluation of frequently visited microenvironments during the winter season. RPMMC was rigorously monitored over an 8 h duration using gravimetric analysis, while RPMNC was continuously assessed for an average of 22 h using a Dylos DC 1100 Pro. Notably, the highest peak of personal exposure to RPMMC was noted in December, which coincided with the low ambient temperatures and poor dispersion conditions typical of winters. Among the varied microenvironments scrutinized, the indoor café exhibited the highest RPMNC exposure due to the café’s adjacent kitchen and the presence of a smoke source (live BBQ station beside the café), whereas the outdoor café displayed the highest RPMMC concentrations. These findings underscore the pivotal role of specific activities within microenvironments in shaping their RPM exposure levels.

It is noteworthy that all measured RPMMC and RPMNC values exceeded the ambient PM_2.5_ National Environmental Quality Standards (NEQS) of Pakistan, and yet remained below the permissible limits set by regulatory bodies such as the Occupational Safety and Health Administration (OSHA) and the American Conference of Governmental Industrial Hygienists (ACGIH). A stronger correlation between RPMMC and RPMNC was observed in controlled microenvironments (R^2^ = 0.56) than in personal exposures (R^2^ = 0.17), highlighting the influence of individual behaviors and environmental transitions on PM exposure. The correlation analysis between the RPMMC and RPMNC data sets revealed a stronger relationship within MEs as compared to personal exposure scenarios. This disparity underscores the complex interplay between individual behaviors and environmental factors in shaping personal exposure levels.

### Mitigation Measures

To mitigate PM exposure, universities should prioritize improving ventilation in high-risk MEs, such as by installing air purifiers and upgrading existing HVAC systems in classrooms, cafés, and hostels. Moreover, implementing cleaner practices, such as cooking with a proper exhaust system, could lower the PM concentrations in community spaces.

A comparative analysis revealed that HVAC-equipped buildings at NUST exhibit significantly lower levels of respirable particulate matter, both in terms of mass concentration (RPMMC) and number count (RPMNC), owing to the use of high-efficiency filtration systems (e.g., MERV 13 or higher) and controlled ventilation rates (6–12 ACH). Demand-controlled systems and routine filter maintenance further contribute to the maintenance of optimal indoor air quality. In contrast, non-HVAC buildings demonstrate higher and more variable RPMMC and RPMNC levels, especially during periods of elevated outdoor pollution. Mitigation in naturally ventilated areas includes strategic window operation during low ambient PM hours, the deployment of portable HEPA purifiers in classrooms, and the incorporation of green barriers near openings. These measures are critical for reducing particulate exposure and improving indoor air quality in educational environments where mechanical ventilation is limited.

This study underscores the importance of prolonged monitoring periods for personal exposure assessments, highlighting the need for institutions to move beyond isolated air quality measurements and implement a holistic, tiered approach to managing indoor environmental health. By focusing on both personal exposure patterns and the role of microenvironments, universities can better protect their students’ well-being. Furthermore, while this study focused on specific microenvironments, future research endeavors could benefit from a more comprehensive approach, encompassing a wider array of settings to provide a holistic understanding of RPM exposure dynamics. Additionally, integrating external sources into the monitoring framework would offer invaluable insights into the nuanced fluctuations observed in personal exposure to respirable particulate matter across different environmental contexts.

## Figures and Tables

**Table 1:** Statistical results of RPMC in µg/m^3^ and RPMNC in m^−3^ of personal exposure of students.

	Mean	Median	Mode	St. dev.	Min	Max	Range	n
RPMMC (PM_4_) (µg/m^3^)	251	245	239	91	43	505	461	40
RPMNC_2.5_ (m^−3^)	175	161	No mode	84	52	483	431	40

RPMNC_2.5_ (m^−3^) data had no mode.

**Table 2:** Monthly mean value of RPMC in µg/m^3^ and RPMNC in m^−3^ of personal exposure of students.

	Nov.	Dec.	Jan.
RPMMC (PM_4_) (µg/m^3^)	242	266	240
RPMNC_2.5_ (m^−3^)	152	236	129

**Table 3:** Statistical results of RPMMC in µg/m^3^ and RPMNC in m^−3^ of personal exposure in all microenvironments.

	Mean	Median	St. Dev.	Min	Max	Range	n
RPMMC (PM_4_)	69.36	63	51	10	187	177	18
RPMNC_2.5_	87	80	1213	31	523	5197	18

## Data Availability

Data will be made available upon request.

## Supplementary Materials

The supporting information for this manuscript can be downloaded at https://www.mdpi.com/article/10.3390/toxics13070571/s1. Figure S1 shows the map of the study site and surrounding areas that mainly contribute to atmospheric PM pollution; Figure S2 depicts the methodology layout of this study; and Figure S3 provides the monthly mean precipitation data for the study months.
