# Supplementary material for: Personal Exposure Assessment of Respirable Particulate Matter Among University Students Across Microenvironments During the Winter Season Using Portable Monitoring Devices
Source: Toxics. 2025 Jul 7;13(7):571. doi: 10.3390/toxics13070571 (PMC12299022; doi:10.3390/toxics13070571)
Supplement: Supplementary file 1 [file toxics-13-00571-s001.zip › toxics-3706867-supplementary.pdf]

Supplementary Information

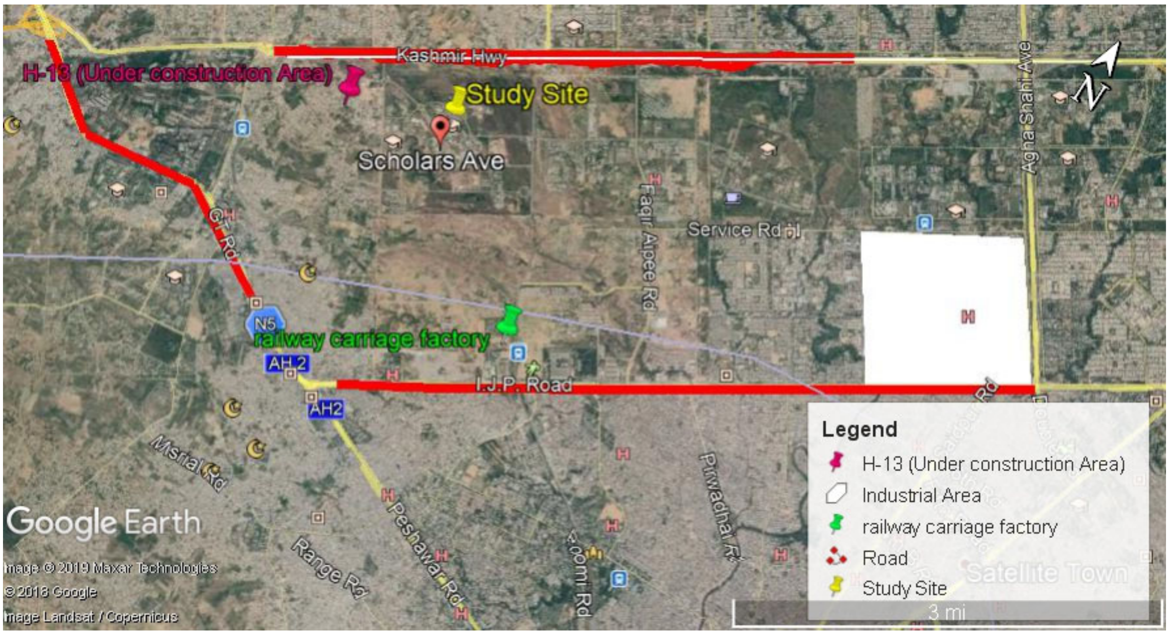

Figure S1: Map of the study site and surrounding areas that mainly contribute to atmospheric PM pollution

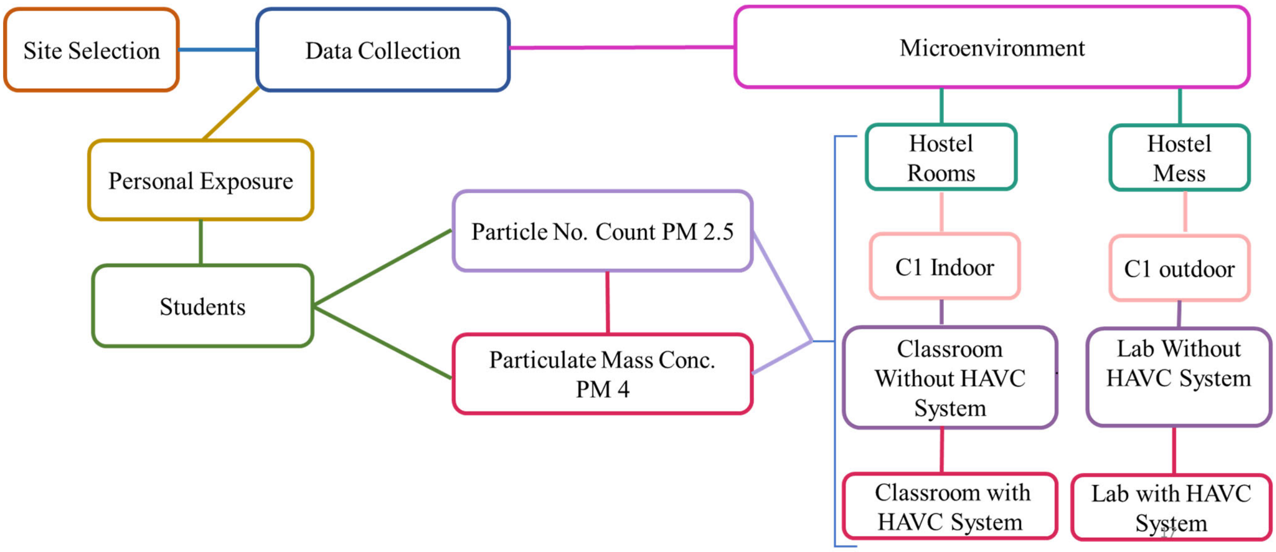

Figure S2: Methodology Layout of the study

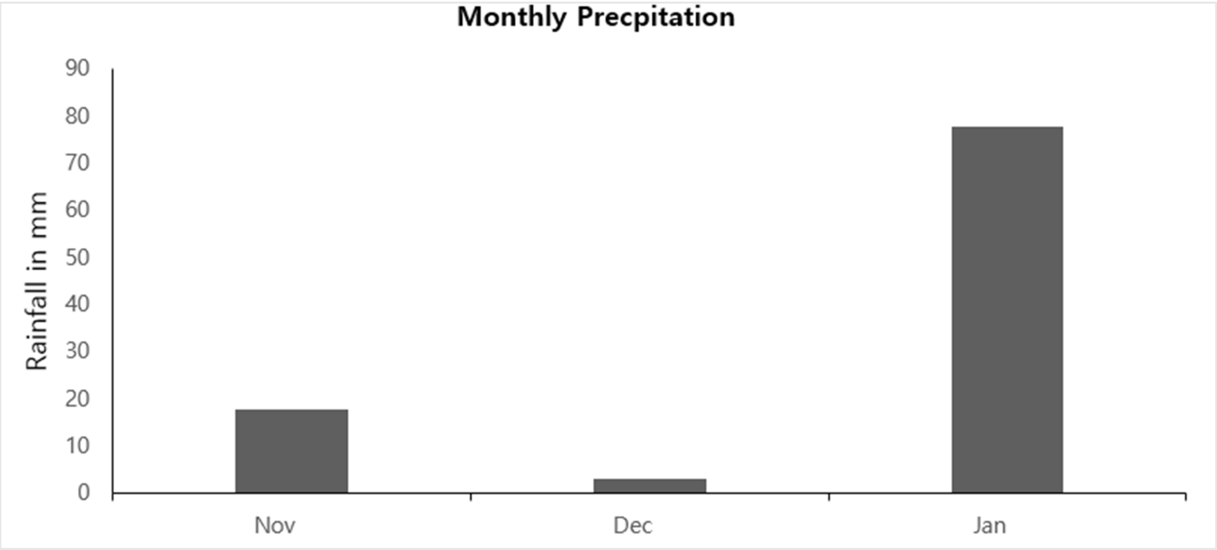

Figure S3: Monthly mean precipitation data
